# Missed diagnosis of lissencephaly after prenatal diagnosis: A case report

**DOI:** 10.1097/MD.0000000000033014

**Published:** 2023-02-17

**Authors:** Mengna Liu, Xiao Liu, Jiebin Wu, Jing Sha, Jingfang Zhai, Bei Zhang

**Affiliations:** a Department of Prenatal Diagnosis Medical Center, Xuzhou Central Hospital, Xuzhou Clinical School of Xuzhou Medical University, Xuzhou, Jiangsu, China; b Department of Pediatrics, Xuzhou Central Hospital, Xuzhou Clinical School of Xuzhou Medical University, Xuzhou, Jiangsu, China; c Key Laboratory of Brain Diseases Bioinformation of Xuzhou Medical University, Xuzhou, Jiangsu, China.

**Keywords:** epilepsy, lissencephaly, PAFAH1B1, whole-exome sequencing

## Abstract

Lissencephaly (LIS) is a rare and serious cortical malformation characterized by a smooth or nearly smooth brain surface. With the progress of molecular genetics, platelet-activating factor acetylhydrolase brain isoform Ib is the most frequent type during the fetal period. Here, we report an infant with LIS who was missed although undergoing prenatal diagnosis. We aim to share our experiences and lessons.

**Patient concerns:**

A 2-month-old male infant presented recurrent convulsions. Karyotype and copy number variation sequencing were conducted to be normal at the 23-week gestation because of bipedal varus and ventricular septal defect (2.3 mm). After birth, he suffered from epilepsy confirmed by video electroencephalogram exam, meanwhile, computed tomography and magnetic resonance imaging revealed pachygyria. The infant was diagnosed with LIS carrying a de-novo mutation c.817 C > T (p.Arg273 Ter,138) in exon 8 of platelet-activating factor acetylhydrolase brain isoform Ib (NM_000430) detected by whole-exome sequencing.

**Diagnoses:**

Based on the clinical characteristics, imaging, and genetic test findings, the infant was diagnosed with LIS.

**Interventions:**

The patient was treated with topiramate and dose was adjusted according to the seizure frequency.

**Outcomes:**

The infant had recurrent seizures. The muscle tone of his extremities increased, and he could not look up or turn over actively at the age of 6 months.

**Lessons:**

Comprehensive evaluation of a multi-disciplinary team should be recommended for patients with epilepsy and cerebral hypoplasia. Individuals with LIS during the fetal period might be missed due to atypical features. In fetuses with structural abnormalities, if karyotype and copy number variation sequencing are both normal, whole-exome sequencing may be an effective complementary means to detect pathogenic variants.

## 1. Introduction

Lissencephaly (LIS) is a serious brain developmental disorder resulting from the arrest or defect of neuronal migration caused by environmental and genetic factors such as gene mutation and early pregnancy infection with an incidence rate of about 1/100000 to 4/100000.^[1–3]^ It is a “smooth brain” characterized saliently by absent or excessively wide gyri, along with cortical thickening, disorganization, and misplaced neurons in the subcortical white matter, including agyria, pachygyria, and gray matter heterotopia.^[4]^ Clinically, patients with LIS mainly present with feeding problems, delayed motor milestones, mental retardation, early epileptic encephalopathy, and other neurological malformations such as hypoplasia of the callosum. Most of them have poor prognoses.

With the rapid progress of molecular genetics, more and more related gene variations have been confirmed. LIS has obvious genetic heterogeneity. Among these variations, deletions of different sizes in 17p13.3 including platelet-activating factor acetylhydrolase brain isoform Ib (PAFAH1B1) or mutations in PAFAH1B1 were the most frequent molecular etiologies.^[5]^ The PAFAH1B1 gene is located on chromosome 17p13.3 and encodes the PAFAH1B1 protein containing 2 catalytic subunits and a regulatory subunit. The regulatory subunit is critically regulated and when deficient leads to the devastating human neurological disorder LIS or smooth brain. The role of the protein in brain development is not the catalysis of platelet-activating factor, rather the entire brain platelet-activating factor acetylhydrolase complex serves a signaling role, coordinating important pathways in brain development. The pathogenic variations of PAFAH1B1 can affect the function of microtubule-associated proteins in neuronal migration, resulting in LIS and autosomal dominant inheritance.^[6]^

In this study, we report an infant with LIS based on the clinical characteristics, imaging, and genetic test findings who was missed after undergoing routine prenatal diagnosis. We aim to share our experiences and lessons about the infant.

## 2. Case presentation

A 2-month-old male infant was hospitalized due to recurrent convulsions for 5 hours. The infant had a sudden onset of clonic seizures without any causes. He was the first child of a non-consanguineous and healthy Chinese couple. At the 25th week of gestation, amniocentesis was conducted due to structural abnormities (bipedal varus and ventricular septal defect [2.3mm]) in ultrasound, and the results of karyotype and copy number variation sequencing (CNV-seq) were both normal. The infant was born by vaginal delivery at 38 weeks and 3 days of pregnancy, weighing 2.56 kg. Apgar scores were 10 and 10 at 1 and 5 minutes respectively. No intrauterine distress or postnatal asphyxia had occurred.

**Figure 1.**
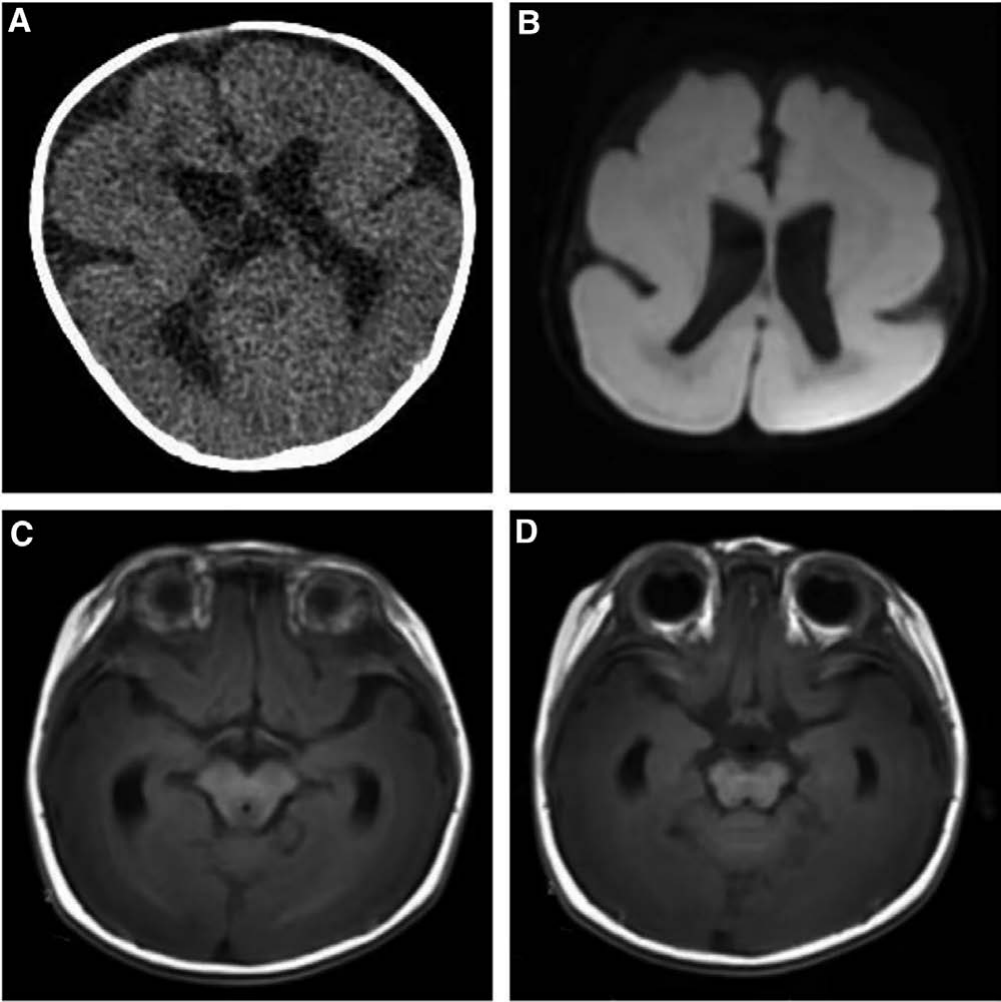
(A) Computed tomography showed enlarged cerebral gyrus and thick cerebral cortex when the infant was 2 months old. (B–D) MRI results at 6-month old showed a diffuse lissencephaly-pachygyria spectrum, enlarged bilateral lateral ventricles, and slightly smaller bilateral hippocampal heads, midbrain as well as pons. MRI = magnetic resonance imaging.

**Figure 2.**
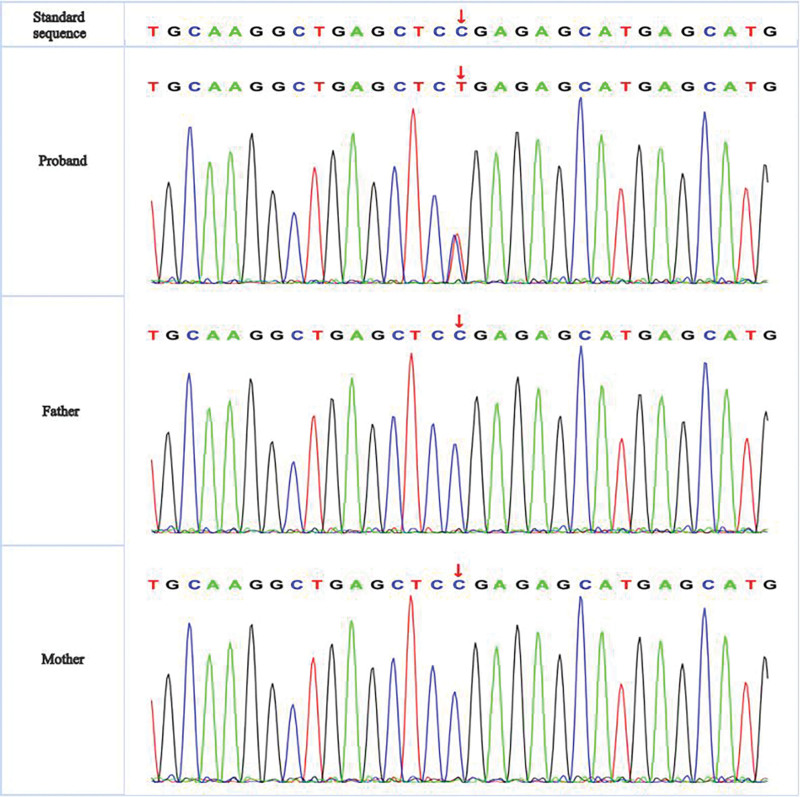
Trio-WES results of this case. (A) De novo pathogenic mutation in PAFAH1B1(exon8:c.817C > T:p.Arg273 Ter,138) of the infant; father and mother present no variant. PAFAH1B1 = platelet-activating factor acetylhydrolase brain isoform Ib, WES = whole-exome sequencing.

The infant was listless and the neck resistance examination was positive. Bipedal varus were not seen. The cytomegalovirus test was negative. Atrial septal defect (3 mm) was detected by heart ultrasound without ventricular septal defect. Video electroencephalograph exam was carried out indicating diffuse activity, mixed with multiple foci and widespread sharp-slow waves and sharp waves. After 5 focal seizures, a series of spastic seizures appeared, supporting the diagnosis of epilepsy. Computed tomography showed that the cerebral gyrus was enlarged and the cerebral cortex was thick (Fig. 1A). He failed the brain magnetic resonance imaging (MRI) examination in his first hospitalization. On the following day, at the age of 6 months, MRI revealed that bilateral cerebral gyrus was significantly thickened with less cerebral sulcus. Bilateral hippocampal heads, midbrain, and pons were slightly smaller. Bilateral lateral ventricles were enlarged (Fig. 1B–D). Thus, the patient was diagnosed with pachygyria. The whole-exome sequencing (WES) results of the infant and his parents showed PAFAH1B1 exon8 NM_000430: c.817 C > T: p.Arg273 Ter,138. The pathogenic mutation in this gene caused the arginine to change into a stop codon which terminated translation in advance resulting in the original protein shortening 138 amino acids, and the variant was confirmed as a de novo mutation with Sanger Sequencing (Fig. 2).

Based on the clinical characteristics, imaging, and genetic test findings, the infant was diagnosed with LIS. The patient was treated with topiramate and the dose was adjusted according to the seizure frequency. During follow-up, the infant had recurrent seizures. The muscle tone of his extremities increased, and he could not look up or turn over actively at the age of 6 months. The patient’s father has provided informed consent for the publication of the case. This study was approved by the institutional ethics committee of Xuzhou Central Hospital (XZXY-LJ-20190210-037).

## 3. Discussion

LIS can usually be prenatally diagnosed at multi-disciplinary Fetal Neurology clinics by multiplanar neurosonography and MRI. The brain MRI features of the children with LIS include a smooth surface of the cerebral hemisphere, absence of sulcus, widened cerebral fissure, and decreased white matter.^[1]^ Because prenatal examination of pregnant women is mainly through ultrasound, ultrasound is the main way to discover fetal brain abnormalities for the first time. The patients are usually referred following prenatal ultrasonic screening. These ultrasonography features usually include commissural anomalies, ventriculomegaly, asymmetric ventricles, absent cavum septum pellucidum, cerebellar vermian and/or hemispheric anomalies, abnormal head circumference, multiple central nervous system formations, and associated systemic defects. Experienced ultrasound doctors can find obvious cerebral cortex abnormalities. But for single cortical dysplasia, it can often be missed, just like this infant. In this case, the fetal ultrasound showed structural abnormalities (ventricular septal defect and bipedal varus). Karyotype and CNV-seq of amniotic fluid cells were performed with negative results. According to the current guideline, no further examination was conducted. Unfortunately, the fetus was missed. In the prenatal diagnosis center, professional neurological ultrasound physicians should be essential. When structural abnormalities are indicated, the nervous system, especially the brain, needs to be evaluated.

After birth, the infant presented early epileptic encephalopathy which was a group of intractable epilepsy that starts from weeks to months after birth. Extensive cognitive function and sensorimotor development disorders can be gradually aggravated by frequent epileptiform discharges. Although its pathogenesis is still under study, it has been known that it is closely related to structural abnormalities.^[7,8]^ Most scholars believe that the abnormal synaptic connections and network structures caused by abnormal neuronal migration increase the local or widespread excitement of the brain. While the weakening or loss of intermediate inhibitory neurons leads to the imbalance of excitation inhibition and the formation of lesions, which is also the main cause of drug-resistant epilepsy.^[9]^ During follow-up, the infant presented recurrent seizures, increased muscle tension in his limbs, and delayed motor milestones even though he was treated with topiramate. Obviously, its prognosis is poor.

The patient’s computed tomography and MRI examination indicated pachygyria. Besides, bilateral hippocampal heads, midbrain as well as pons were slightly smaller and bilateral lateral ventricles were enlarged. Many genetic variants have been reported to be associated with it; furthermore, WES was performed to show a de novo gene mutation of exon 8 in PAFAH1B1, resulting in the amino acid change. This variant is inherited as an autosomal dominant trait, and its associated clinical characteristics include mental retardation, seizures, general growth stunt, spastic quadriplegia, gyral hypertrophy, cerebellar hypoplasia, agyria, pachygyria, brainstem dysplasia, brain white matter abnormality, acquired microcephaly, trunk muscle hypotonia, perivascular space, and subcortical zonal heterotopia.^[5,10]^ Therefore, the harmfulness of PAFAH1B1 gene mutation, in this case, is related to the patient’s phenotype.

From this case, we deem that you can carefully inquire about the medical history and family history when structural abnormalities are found in 2 or more systems by ultrasound. If necessary, you can further complete an MRI examination so as to timely recognize the abnormal development of the cerebral cortex that may be missed by ultrasound, and provide strong evidence for the consultation of the eugenics clinic. Besides, WES can often be used as an effective complementary means to effectively detect pathogenic variants in prenatal diagnosis cases with structural abnormalities by ultrasound examination and with normal karyotype and CNV-seq, so as to improve the diagnostic rate of prenatal diagnosis.

## Acknowledgments

We would like to thank the participation of the infant’s parents and the staff’s cooperation with the Departments of Prenatal Diagnosis Medical Center and Pediatrics of Xuzhou Central Hospital.

## Author contributions

**Conceptualization:** Mengna Liu, Jingfang Zhai, Bei Zhang.

**Data curation:** Mengna Liu, Xiao Liu, Jiebin Wu.

**Formal analysis:** Mengna Liu, Xiao Liu, Jingfang Zhai.

**Funding acquisition:** Mengna Liu, Jingfang Zhai.

**Resources:** Jingfang Zhai.

**Supervision:** Xiao Liu, Jiebin Wu, Jing Sha, Jingfang Zhai, Bei Zhang.

**Validation:** Xiao Liu, Jing Sha.

**Writing – original draft:** Mengna Liu.

**Writing – review & editing:** Jingfang Zhai.
